# The reciprocal effect of pain catastrophizing and satisfaction with participation in the multidisciplinary treatment of patients with chronic back pain

**DOI:** 10.1186/s12955-015-0359-5

**Published:** 2015-09-30

**Authors:** Erik Farin

**Affiliations:** Institute for Quality Management and Social Medicine, University of Freiburg - Medical Center, Engelbergerstr. 21, D-79106 Freiburg, Germany

**Keywords:** Pain catastrophizing, Social participation, Low back pain, Multidisciplinary pain treatment, Cross-lagged models

## Abstract

**Background:**

The aim of the study was to examine the reciprocity between pain catastrophizing, social participation and quality of life outcomes (pain intensity, pain disability, negative affectivity) in patients with low back pain in a multidisciplinary pain treatment.

**Methods:**

Patients undergoing inpatient rehabilitation were surveyed at the beginning and two weeks after the end of rehabilitation. *N* = 262 low back pain patients participated (mean age: 52.2, 62.1 % female). A two-wave cross-lagged design and structural equation modeling were used to analyze data.

**Results:**

We found evidence of reciprocal relations with regard to several outcomes. For example, pain catastrophizing at the beginning of treatment is associated with negative affectivity after rehabilitation, and the post-treatment value of pain catastrophizing is associated with pain disability and satisfaction with participation at the start of treatment. Pain disability and pain catastrophizing are predictors of lower treatment outcome while pain intensity and negative affectivity are not risk factors. Participation stands in a reciprocal relationship with some of the pain treatment outcomes. The surprising result, namely, that those patients more satisfied with social participation experience less improvement regarding catastrophizing, can be explained by ceiling effects and the Communal Coping Model.

**Conclusions:**

This study provides evidence of the importance of taking reciprocal relations among pain catastrophizing, social participation and other pain outcomes into account. Providers of multidisciplinary pain treatment need to play attention to patients at risk with high disability and catastrophizing thoughts. Pain treatment would benefit from closer integration of psychosocial measures to foster social participation.

## Introduction

Multidisciplinary chronic pain treatment combines elements of medical management, cognitive-behavioral therapy, physical therapies and occupational therapies. Although knowledge of correlates of treatment outcomes is important to optimize therapeutic measures and test cognitive-behavioral models of chronic pain, little research has examined predictors of change in quality of life outcomes after multidisciplinary chronic pain treatment [1, 2]. Factors assumed to be associated with changes in pain, function and disability, are cognitive and coping variables [2–7].

A weakness of many studies is that they failed to consider the reciprocity of relationships between cognitive factors on the one hand and treatment outcomes on the other. Many only analyze whether cognitive variables influence pain and disability, but not whether pain outcomes have an effect on cognition. Reciprocal relationships are, however, plausible in many respects, and have in fact been proven empirically [3, 6, 8–10].

Aim of this study was to examine this reciprocity with regard to pain catastrophizing in patients with low back pain undergoing multidisciplinary pain treatment. Pain catastrophizing is conceptualized as a negative cognitive–affective response to pain and has been associated with a number of important pain-related outcomes including clinical pain severity, pain-related activity interference, disability and alterations in social support [11, 12]. Sub-aspects of pain catastrophizing discussed in the literature include helplessness, pessimism, magnification of the threat value of pain and rumination. Mechanisms by which pain catastrophizing might be associated with adverse outcomes include schema-activation models, attention and information processing biases, appraisal models, and endogenous pain modulation pathways. The Communal Coping Model [13, 14] focuses on social behavioral dimensions of catastrophizing and opposes an overly “individualistic” conceptualization of pain coping processes.

The distinction between pain catastrophizing and negative affectivity in general (e.g., neuroticism, depression, anxiety) has often been explored [3, 11, 15–17], which is why we devoted particular attention to it in this study.

Another aim of this study was to examine the relevance of a variable that has attracted little attention in this context, namely social participation. In health care research there is growing interest in a person’s participation [18], as well as the individual’s involvement in life situations in relation to health conditions, body functions and structure, activities, and contextual factors [19]. Social participation plays a key role within the Communal Coping Model. If individuals differ in the degree to which they adopt relational goals in their efforts to cope with pain, it would seem to follow that satisfaction with social participation is associated with pain coping and pain outcomes.

Three hypotheses were examined in this study:

1) There is a reciprocal relationship between pain catastrophizing and the pain treatment outcomes considered herein (pain intensity, pain disability, satisfaction with participation, negative affectivity). This means that pain catastrophizing at the start of multidisciplinary chronic pain treatment (t0) is associated with pain outcomes after treatment (t1), just as the pain outcome values at the beginning of therapy are associated with pain catastrophizing afterwards. We will analyze in an exploratory way to which of the pain outcomes this applies.

2) The predictive association of pain catastrophizing at t0 with pain treatment outcomes is higher than that of negative affectivity at t0.

3) There is a reciprocal relationship between satisfaction with participation and the pain treatment outcomes addressed herein. Again, we will analyze in an exploratory way to which of the pain outcomes this applies.

## Materials and methods

### Participants

Patients with chronic low back pain undergoing inpatient rehabilitation were surveyed. The study was approved by the Ethics Committee of the University of Freiburg (approval number 23/09). In Germany, the goal of multidisciplinary pain treatment in rehabilitation is to prevent or mitigate the impairment of participation in working and social life. Examples of individual treatment elements are training based on a biopsychosocial disease model, occupational therapy, physical therapy, exercise therapy, education, and psychotherapeutic treatment to modify maladaptive illness behavior and learn techniques for coping with stress. Inpatient rehabilitation is standardized in terms of the therapeutic measures taken as well as their lengths. Rehabilitation generally lasts 3 weeks and is oriented around current guidelines and therapeutic standards (i.e., the National Guideline for Back Pain, Rehabilitation Therapy Standard for Chronic Back pain [20, 21]).

The criterion for inclusion in our study was chronic low back pain for at least 6 months. Patients with specific low back pain due to tumors or inflammatory diseases were excluded. Patients were approached by their clinic’s personnel at the beginning of rehabilitation and asked to fill out a self-administered paper-and-pencil questionnaire at the beginning and two weeks after the end of rehabilitation. These were given only to patients able and willing to fill them out (informed consent). Of the 374 patients asked to participate in the study, 266 (71.1 %) consented.

Several individuals had to be excluded because of implausible data, thus the final sample contained *N* = 262 patients. The most frequent reason for study non-participation was unwillingness (43.5 %), followed by physical or cognitive impairment (9.3 %), and a lack of adequate German language skills (6.5 %). 40.7 % of the eligible patients provided no reason for non-participation. The dropout rate two weeks after the end of rehabilitation was 16.4 %. Table 1 provides an overview of some characteristics of our sample.

**Table 1:** Respondent characteristics (*N* = 262)

Sociodemographic characteristics	
Age (Mean/SD)	52.2 (10.1)
Sex	
% female	62.1
Regular partner	
% yes	68.8
Level of education (highest level completed)	
% elementary school	28.1
% secondary school	43.4
% university-entrance diploma or technical college qualification	24.6
Employment	
% employed	80.1
Medical and pain characteristics	
Pain intensity (VAS 0–100, Mean/SD)	54.0 (23.4)
SF-36: Bodily pain (Mean/SD)	36.9 (20.3)
Duration of disease (%)	
<1 year	10.8
1–2 years	14.7
3–5 years	23.6
6–10 years	23.6
>10 years	26.3
Psychological characteristics	
HADS: Anxiety (% conspicuous values^1^)	31.8
HADS: Depression (% conspicuous values^1^)	19.2

^1^scale values above 11

### Measures

To measure pain catastrophizing, the Pain Catastrophizing Scale PCS was used [22] (German version [23]) which contains three subscales assessing rumination, magnification and helplessness. Satisfaction with social participation was assessed by applying a static form of the PROMIS® Item Bank for Satisfaction with Participation [24] (German version [25]). The German version contains two subscales having 13 and 10 Items, respectively (Satisfaction with Participation in Social Roles SP-SR and Satisfaction for Participation in Discretionary Social Activities SP-DSA). Pain intensity was evaluated by applying two variables, namely a 0–100 visual analogue scale (VAS) to assess the intensity of pain (0 = no pain, 100 = extreme pain) and a rating item from the SF-36 (pain in last week, six response categories). The Oswestry Disability Index (ODI [26], German version [27]) and Pain Disability Index (PDI [28], German version [29]) were used as indicators of pain disability. Finally, to operationalize negative affectivity, we referred to the Hospital Anxiety and Depression Scale (HADS [30], German version [31]). All these variables were measured at both time points.

Sociodemographic characteristics of the patients (age, gender, regular partner, highest level of education completed, employment) were recorded using patient or physician information. Medical variables considered were the duration of the illness, treatment motivation assessed by the physician (6-point rating scale, 1 = no motivation, 6 = very high motivation) and comorbidity (measured using a rehabilitation-specific comorbidity score [32]).

### Statistical analysis

A two-wave cross-lagged design and structural equation modeling (SEM) were used to analyze data. This procedure allows us to examine the hypothesized reciprocal associations in the same model and simultaneously control for covariates. Our SEM analyses were conducted using the IBM SPSS AMOS 21 program, on the basis of the maximum likelihood estimation procedure. We attend to the problem of missing data by using multiple imputation with SAS 9.2 PROC MI and MIANALYZE [33]; see also recommendations by Mayer et al. [34]. Following Rubin [35], five imputed data sets were created. The relevant parameters were combined according to Rubin’s rules [35].

The SEM analyses were conducted in several stages:

First, we tested the measurement model according to the method suggested by Cole and Maxwell [36]. Multiple indicators for each latent variable were used. Pain catastrophizing, satisfaction with participation and negative affectivity were indicated by the subscales of PCS, PROMIS® and HADS, respectively. Pain intensity was indicated using the VAS and global pain item. Finally, pain disability was indicated by ODI and PDI. The measurement model is tested via confirmatory factor analysis (CFA) that estimates the manifest indicators’ loadings on their respective latent variables.

We tested a model in which every latent variable is allowed to correlate with every other latent variable [36]. The error terms of each indicator at t0 and t1 were allowed to covary with each other, as is the recommended procedure in longitudinal structural equation models [37, 38]. To set the scale of the latent variables, one factor loading per latent variable was set to unity.

To establish the measurement model, statistically significant loadings, as well as an acceptable model fit, are required. Model fit was evaluated using the Comparative Fit Index (CFI [39]), Tucker-Lewis index (TLI [40]), root mean square error of approximation (RMSEA), and standardized root mean square residual (SRMR). CFI and TLI values >0.90 are an indication of good fit. RMSEA values <0.10 suggest a moderate fit; values <0.05 are a good fit [39]. The SRMR value should be under 0.08 [39]. Sufficient model fit is assumed whenever at least three of four parameters produce good values [41].

In the second step, CFA was performed to test for (weak) factorial invariance across measurement waves. The baseline measurement model was compared to a more constrained model, including equality of factor loadings over the two assessment points. In accordance with other studies [42, 43] a decrease in CFI of more than .01 was considered representative of a decrease in model fit indicating that the additional constraints imposed on the model (compared with the previous model) cannot be justified.

**Fig. 1:**
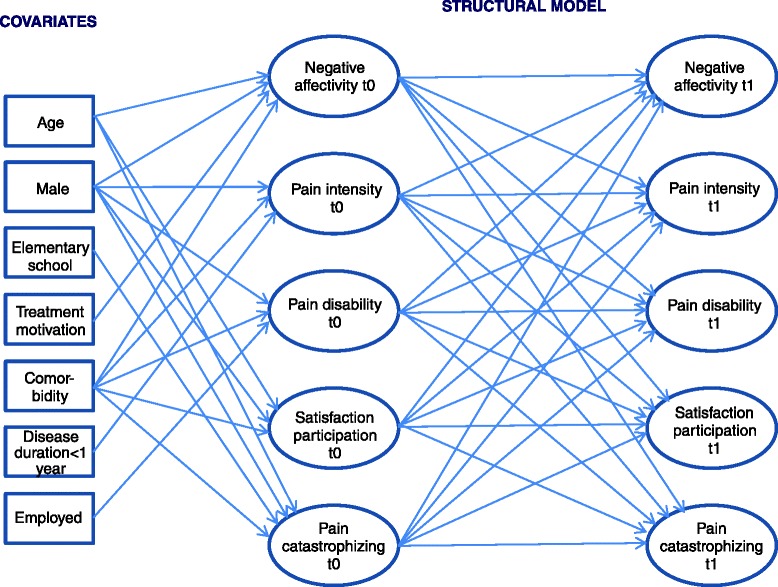
Cross-lagged model with reciprocal relations between all latent variables that was tested in the third step. Note: The model is illustrated here in simplified form (without indicators of latent variables and without error terms). The error terms of each indicator at t0 and t1 were allowed to covary with each other. Furthermore, synchronous correlations between error terms in the same wave were allowed

In the third step a cross-lagged model with reciprocal relations between all latent variables was tested. As sociodemographic and medical covariates of baseline values we included age, gender, education, employment, treatment motivation, comorbity and duration of the illness. For reasons of parsimony, however, insignificant paths (*p* > = .05) were deleted. Synchronous correlations between error terms in the same wave were allowed. Auto-regression effects were included to control for baseline levels. The tested model is presented in Fig. 1.

In the next step all insignificant cross-lagged paths (*p* > = .05) were removed. Finally, all remaining cross-lagged paths were checked. A path was retained if the model fit of the model without this path was significantly worse than the model’s fit including this path. To test this, we first conducted a nested-model comparison based on the chi-square difference test [44].

## Results

Before proceeding to the SEM results, Table 2 describes the changes between t0 and t1 in all the relevant variables. Significant positive changes with medium effect sizes after rehabilitation were generally achieved. Negative affectivity revealed weaker effects, while they were stronger with pain intensity. The effect size of reduction in pain catastrophizing was low to medium (0.24–0.35). The measurement model fits well (see Table 3); all the fit indices fell into the required range. The manifest variables’ loading on their respective constructs consistently exceeds 0.78. Weak factorial invariance across measurement waves exists; the assumption that the factor loadings would be the same at the two assessment points was confirmed (Table 3). The model tested in step three is displayed in Fig. 1 in somewhat simplified form. The fit here was also good (Table 3). In the next step, all insignificant cross-lagged paths were removed. Five cross-lagged paths remained:

- pain disability t0 → negative affectivity t1
- pain disability t0 → satisfaction participation t1
- pain disability t0 → pain catastrophizing t1
- satisfaction participation t0 → pain catastrophizing t1
- pain catastrophizing t0 → negative affectivity t1

**Table 2:** Changes between beginning of rehabilitation (t0) and two weeks after the end of rehabilitation (t1)

	t0	t1	t	Effect size
	(Mean, Standard deviation)	(Mean, Standard deviation)	(*p* value)	
Pain Catastrophizing				
Rumination (PCS-R)	8.36 (4.81)	6.70 (5.28)	5.51 (<.001)	0.35
Magnification (PCS-M)	4.17 (2.74)	3.51 (2.93)	4.40 (<.001)	0.24
Helplessness (PCS-H)	7.63 (4.77)	5.96 (5.11)	6.77 (<.001)	0.35
Satisfaction with Participation				
Satisfaction with Participation in Social Roles (SP-SR)	−0.72 (3.16)	0.42 (3.39)	−5.70 (<.001)	0.36
Satisfaction with Participation in Discretionary Social Activities (SP-DSA)	−1.18 (2.94)	0.26 (3.29)	−6.88 (<.001)	0.49
Pain intensity				
Pain VAS	53.6 (23.1)	35.8 (24.1)	9.80 (<.001)	0.77
Pain item	4.15 (1.03)	3.49 (1.24)	8.42 (<.001)	0.64
Pain disability				
ODI	35.9 (17.3)	28.9 (17.8)	8.43 (<.001)	0.40
PDI	33.1 (16.8)	25.8 (17.4)	7.72 (<.001)	0.43
Negative affectivity				
Depression (HADS-D)	6.29 (4.19)	5.68 (4.71)	2.41 (.017)	0.15
Anxiety (HADS-A)	8.01 (4.26)	6.53 (4.62)	5.77 (<.001)	0.35

Note: N_min_ = 136, N_max_ = 216. Effect size was calculated as mean difference standardized by the standard deviation at t0. The range of PCS-R and PCS-H is 0 to 20, the range of PCS-M 0 to 12. Higher values indicate greater catastrophizing. The range of SP-DSA is -6.79 to 8.43, of SP-SR -7.09 to 8.58. Higher values stand for better participation. High values regarding the two indicators for pain intensity stand for stronger pain. The range of ODI is 0-100, that of PDI 0-70. Higher values represent greater pain disability. The range of both HADS scales is 0–21, with higher values reflecting more negative affectivity

**Table 3:** Fit of models

	Chi^2^	df	CFI	TLI	RMSEA	SRMR
Step 1: Measurement model	348.22	153	0.969	0.954	0.070	0.037
Step 2: Weak factorial invariance	378,34	164	0.966	0.953	0.071	0.045
Step 3: Cross-lagged model with reciprocal relations between all latent variables	697,28	311	0.942	0.924	0.069	0.072
Step 4: Final cross-lagged model	739,50	326	0,938	0,923	0,070	0,083

**Fig. 2:**
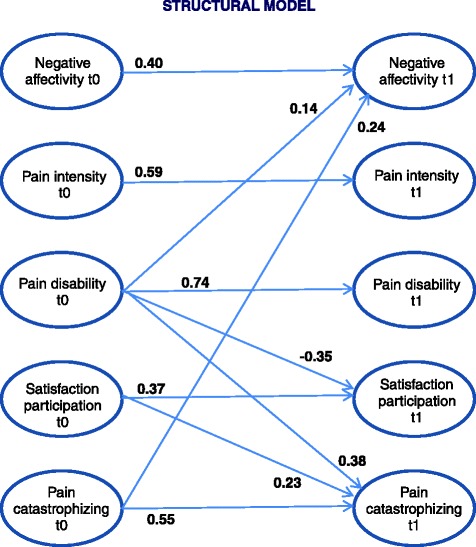
Structural model of the cross-lagged model with standardized regression weights

The final cross-lagged model’s fit was also good (Table 3). All five paths proved to be significant. The model without the respective path demonstrated a significantly worse model fit (*p* < 0.05). Figure 2 visualizes this finding by displaying the structural model’s path coefficients.

Hypothesis 1 was confirmed, as pain catastrophizing at the start of pain treatment is associated with negative affectivity thereafter on the one hand and on the other, the post-treatment value of pain catastrophizing is associated with pain disability and satisfaction with participation at the start of treatment. However, this reciprocity only applies to the aforementioned outcomes, not all of the outcomes that were examined. The relationships are directed as one would expect, namely that back pain patients who tend to have catastrophizing thoughts at the beginning of the intervention display greater negative affectivity after treatment. More severe disability and less satisfaction with participation at the start of treatment are associated with more catastrophizing thoughts thereafter.

Hypothesis 2 was also confirmed, as pain catastrophizing at t0 represents a predictor of negative affectivity at t1, while negative affectivity at t0 is not relevant to any other outcome variable.

Satisfaction with participation also revealed a reciprocal relationship with the pain treatment outcomes addressed herein. For one, satisfaction with participation at t0 is associated with pain catastrophizing at t1. Furthermore, satisfaction with participation after rehabilitation is influenced by pain disability at t0. While the latter relationship fulfills our expectation (with greater disability being a risk factor), it is the positive association between satisfaction with participation at t0 and pain catastrophizing at t1 that is surprising.

Table 4 illustrates the covariates’ roles in the final cross-lagged model. Comorbidity is the most important risk factor. Moreover, men and older patients present more favorable pain variable values at the start of treatment.

**Table 4:** Significant paths between covariates and baseline values

	Negative affectivity	Pain intensity	Pain disability	Satisfaction with participation	Pain catastrophizing
Age	−0.15**	-	-	-	−0.13**
Gender: male	−0.14*	−0.19**	-	0.13*	−0.20***
Comorbidity	0.30***	0.30***	0.33***	−0.23***	0.39***
Duration of disease: <1 year	-	−0.15***	-	-	-
Employment	-	-	−0.14***	-	-

Standardized regression weights and significance (*p < .05, **p < .01, ***p < .001)

## Discussion

### The reciprocal relation between pain catastrophizing and pain treatment outcomes

We found evidence of a reciprocal relationship. However, the question remains as to why pain catastrophizing in this study was only associated with negative affectivity after treatment and not with other outcomes. This finding appears to contradict other studies addressing the significance of pain catastrophizing. Yet it is important to keep in mind which hypotheses were tested with our cross-lagged model. There are two possible reasons why pain catastrophizing could be irrelevant to changes in pain disability or pain intensity after treatment: 1) that pain catastrophizing plays no role in the process of change, or 2) that treatment providers successfully compensate for the negative influence of pain catastrophizing during therapy, i.e. by specifically addressing catastrophizing thoughts in patients who are especially incapacitated. Thus there is not necessarily a discrepancy between this finding and those from working groups who investigated mechanisms of change and demonstrated that changes in catastrophizing are associated with improvements in pain outcome [3, 5, 6, 8, 45]. We provide evidence that, when considering reciprocal relationships and adjusting numerous other variables, pain catastrophizing do not represent a risk factor for the outcome of a multidisciplinary pain treatment. As there is ample evidence of the significance of pain catastrophizing regarding the process of change, we suspect that explanation 2) above is the correct one, that is, that the negative effect of pain catastrophizing is alleviated during rehabilitation therapy. That this does not apply to negative affectivity as well may have to do with the fact that that aspect is not generally a major aim of pain treatment in rehabilitation. In Germany, rehabilitation focuses on improving activities and participation. Another potential or supplemental explanation is that pain catastrophizing is such an obvious risk factor for negative affectivity that it is hard to neutralize its effect by therapeutic interventions.

Our results are in line with findings made by Kovac et al. [29], who also observed that catastrophizing did not predict low back pain or disability 3 months after routine clinical practice. In their study of similar design and after controlling for other psychological variables, Foster et al. [46] found that in a primary-care setting and large patient cohort, pain catastrophizing was not significantly associated with disability. Yet there are a few studies (as in the recent systematic review by Wertli et al. [47]) that provide evidence of an influence of catastrophizing on treatment outcome in patients with low back pain. Drawing a detailed comparison with our results would be difficult because very few studies have addressed the reciprocity of relationships between cognition and disability and because the interventions under investigation differ so strongly.

Most of the studies examining over time and without a specific intervention whether pain catastrophizing in chronic pain patients is a predictor for future outcomes such as pain intensity or pain disability have detected an effect [11, 48]. Nevertheless, they cannot be directly compared with the present findings, which refer to change after a specific pain intervention. There is some evidence [46, 49] that catastrophizing exerts greater influence on the progression of pain in the acute stage than on treatment outcome in a chronic pain cohort.

While pain catastrophizing at t0 was only associated with the outcome negative affectivity, pain catastrophizing after treatment is considerably influenced by pain disability and satisfaction with participation at t0. Pain disability reveals the direction of effect we would anticipate, namely that those persons who are more seriously incapacitated experience less improvements with regard to catastrophizing. Greater disability might have had a negative effect on performing activities during therapy, resulting in a higher level of pain catastrophizing. This finding also seems to contradict results from longitudinal studies reporting that early-treatment reductions in disability were not associated with changes in late-treatment catastrophizing (i.e. [3, 8]). Yet we must remember that a predictor of change after an intervention need not be the causal agent of change.

In contrast to other findings (e.g., Wade et al. [50]), we found that greater pain intensity at t0 was not a predictor for more catastrophizing thoughts. This may have to do with the fact that Wade et al. did not include an assessment of pain disability. Pain disability may be the key variable and we know that the influence of pain intensity is largely mediated by disability. In their review, Crombez et al. [51] conclude that pain interference, not pain itself, may be the key trigger of cognitive, behavioral, and emotional responses to pain.

### The reciprocal relation between satisfaction with participation and pain treatment outcomes

There are also some reciprocal relationships regarding satisfaction with participation. What is surprising, however, is that persons who are more satisfied with participation, experience less improvements in catastrophizing. Perhaps, the Communal Coping Model of pain catastrophizing [13] in combination with the ceiling effect phenomenon may help us to explain this finding. Data from the present study reveal at t0 (not presented in the results section) a substantial and highly significant correlation between satisfaction with participation and pain catastrophizing. For satisfaction with participation in social roles, that correlation amounted to (depending on the PCS scale) -0.35 und -0.51, and -0.29 bis -0.44 for satisfaction with participation in discretionary social activities (*p* < 0.001). Those who are dissatisfied with their social participation tend to experience considerably more catastrophizing thoughts. This finding can be explained by referring to the Communal Coping Model, which postulates that high pain catastrophizers might engage in exaggerated pain expression in order to maximize proximity and empathic responses from others [12]. Persons less satisfied with social participation probably have a greater need for positive contact with others and are thus more apt to make use of the interpersonal function of pain expression. Furthermore, our data (not presented in the results section) illustrate that persons whose satisfaction with participation values at t0 lay above the median already have a more positive pain catastrophizing value at the start of therapy than the t1 mean value in the whole sample. It is conceivable that such a low value leaves us with no leeway for further improvement, which is reflected in the negative correlation between satisfaction with participation at t0 and change in pain catastrophizing.

The result that pain disability at t0 was a predictor for less satisfaction with participation at t1 meets the expectation. Whoever is impaired in carrying out activities is also less apt to engage in social participation, which in turn results in less satisfaction (unless the patient’s expectations and demands have been lowered while coping with his or her disease).

### Pain catastrophizing and negative affectivity

Pain catastrophizing shares significant variance with the broader negative affect construct [11, 16], thus many study groups have discussed the differentiation between these two variables. We postulated that the significance of pain catastrophizing at t0 for pain treatment outcomes is greater than the significance of negative affectivity. This hypothesis has been confirmed in part: pain catastrophizing at t0 is a risk factor for high negative affectivity at t1, while negative affectivity at t0 is not a risk factor for any of the outcomes considered in this research. This argues in favor of the uniqueness of the catastrophizing construct, and corresponds with other studies” results revealing catastrophizing’s influence on outcomes despite having adjusted for negative affectivity [3, 5–7, 17]. The present finding is also in line with the fear-avoidance model [52] as well as with more recent models for pain adaptation (i.e. [53]). However, other study groups have observed that catastrophizing accounted for minimal variance in pain outcomes beyond negative affectivity [16]. Those investigations generally employed, however, only a cross-sectional design, making them not comparable with our approach.

The strengths of our investigation are the use of a cross-lagged structural equation model that takes reciprocal relations and measurement error into account, that multiple pain variables were assessed simultaneously and that the satisfaction with social participation construct seldom tested in pain research was included. On the other hand, the study’s limitations include the fact that only self-reporting measures were applied, meaning that the associations found may be explained, at least in part, by shared method variance [54]. Furthermore, we need to bear in mind that the cross-lagged panel design is still a correlational one. Conclusions about whether the predictors actually cause favorable outcome cannot be drawn from such a design.

Another limitation of the design is that we only have two time points. As a consequence of this, we cannot investigate the influence of *changes* in catastrophizing (or other potential mediator variables) on pain outcomes. In terms of the representativeness of our cohort, the fact that we drew data solely from patients undergoing inpatient rehabilitation in Germany is also a limitation. We cannot know whether these findings will also apply to the healthcare system in other countries or to outpatient rehabilitation.

In summing up, we believe we can state with confidence that this study provides evidence of the importance of taking reciprocal relations among pain catastrophizing, social participation and other pain outcomes into account. Distinguishing between outcomes that represent dependent variables and predictors that represent independent variables is often an artificial endeavor and when it is done, the rationale must be stated. Cross-lagged models that portray reciprocal relations are thus the method of choice. We have demonstrated that pain disability and pain catastrophizing at the start of treatment, are predictors of less therapeutic success, while pain intensity and negative affectivity, on the other hand, are not risk factors.

The practical implication of our findings is that clinicians should pay closer attention to the risk group presenting high disability and high catastrophizing thoughts at start of pain treatment. As these factors are associated with comorbidity, female gender and unemployment (Table 4), these characteristics can be regarded as further indicators of risk. In contrast to sociodemographic variables catastrophizing is susceptible by cognitive-behavioral treatment approaches [55]. Treatment components may include discussing the relation between stress and pain, identifying catastrophizing thoughts, exploring the utility of catastrophizing as a coping response and replacing catastrophizing thoughts [56]. The surprisingly close association between satisfaction with participation and catastrophizing is compatible with the Communal Coping Model and makes us suspect that pain treatment would benefit from more intense integration of psychosocial measures to encourage participation. Detailed studies addressing the relations among social participation, catastrophizing and pain outcomes would be worthwhile.

## References

[1] Burns JW, Day MA, Thorn BE (2012). Is reduction in pain catastrophizing a therapeutic mechanism specific to cognitive-behavioral therapy for chronic pain?. Translat Behav Med.

[2] Jensen MP, Turner JA, Romano JM (2001). Changes in beliefs, catastrophizing, and coping are associated with improvement in multidisciplinary pain treatment. J Consult Clin Psychol.

[3] Burns JW, Kubilus A, Bruehl S, Harden RN, Lofland K (2003). Do changes in cognitive factors influence outcome following multidisciplinary treatment for chronic pain? A cross-lagged panel analysis. J Consult Clin Psychol.

[4] Cassidy EL, Atherton RJ, Robertson N, Walsh DA, Gillett R (2012). Mindfulness, functioning and catastrophizing after multidisciplinary pain management for chronic low back pain. Pain.

[5] Jensen MP, Turner JA, Romano JM (2001). Changes in beliefs, catastrophizing, and coping are associated with improvement in multidisciplinary pain treatment. J Consult Clin Psychol.

[6] Smeets RJEM, Vlaeyen JWS, Kester ADM, van der Heijden GJMG, van Geel ACM, Knotterus JA (2006). Reduction of pain catastrophizing mediates the outcome of both physical and cognitive-behavioral treatment in chronic low back pain. Journal of Pain.

[7] Spinhoven P, ter Kuile M, Kole-Snijders AMJ, Mansfeld MH, den Ouden DJ, Vlaeyen JWS (2004). Catastrophizing and internal pain control as mediators of outcome in the multidisciplinary treatment of chronic low back pain. Eur J Pain.

[8] Burns JW, Glenn B, Bruehl S, Harden RN, Lofland K (2003). Cognitive factors influence outcome following multidisciplinary chronic pain treatment: a replication and extension of a cross-lagged panel analysis. Behav Res Ther.

[9] Kovacs FM, Seco J, Royuela A, Corcoll-Reixach J, Pena-Arrebola A (2012). The prognostic value of catastrophizing for predicting the clinical evolution of low back pain patients: a study in routine clinical practice within the Spanish National Health Service. Spine Journal.

[10] Turner JA, Mancl L, Aaron LA (2004). Pain-related catastrophizing: a daily process study. Pain.

[11] Quartana PJ, Campbell CM, Edwards RR (2009). Pain catastrophizing: a critical review. Expert Rev Neurother.

[12] Sullivan MJLP, Thorn BP, Haythornthwaite JAP, Keefe FP, Martin MP, Bradley LAP (2001). Theoretical perspectives on the relation between catastro-phizing and pain. Clin J Pain.

[13] Sullivan MJL (2012). The communal coping model of pain catastrophising: Clinical and research implications. Can Psychol.

[14] Sullivan MJL, Adams H, Sullivan ME (2004). Communicative dimensions of pain catastrophizing: social cueing effects on pain behaviour and coping. Pain.

[15] Linton SJ, Nicholas MK, MacDonald S, Boersma K, Bergbom S, Maher C (2011). The role of depression and catastrophizing in musculoskeletal pain. Eur J Pain.

[16] Hirsh AT, George SZ, Riley JL, Robinson ME (2007). An evaluation of the measurement of pain catastrophizing by the coping strategies questionnaire. Eur J Pain.

[17] Arnow BA, Blasey CM, Constantino MJ, Robinson R, Hunkeler E, Lee J, Fireman B (2011). Catastrophizing, depression and pain-related disability. Gen Hosp Psychiatry.

[18] Eyssen IC, Steultjens MP, Dekker J, Terwee CBA (2011). Systematic review of instruments assessing participation: Challenges in defining participation. Arch Phys Med Rehabil.

[19] World Health Organization (WHO) (2001). The International Classification of Functioning.

[20] Farin E, Glattacker M, Jäckel WH (2011). Leitlinien und Leitlinienforschung [Guidelines und guideline research]. Bundesgesundheitsblatt Gesundheitsforschung Gesundheitsschutz.

[21] Weinbrenner S, Conrad S, Weikert B (2010). 7 Jahre Nationale VersorgungsLeitlinien (NVL) - Quo vadis? [After seven years of National Disease Management Guidelines: Quo vadis?]. Z Evid Fortbild Qual Gesundhwes.

[22] Sullivan MJL, Bishop SR, Pivik J (1995). The Pain Catastrophizing Scale: Development and validation. Psychol Assess.

[23] Meyer K, Sprott H, Mannion AF (2008). Cross-cultural adaptation, reliability, and validity of the German version of the Pain Catastrophizing Scale. J Psychosom Res.

[24] Bode RK, Hahn EA, DeVellis R, Cella D, on behalf of the Patient-Reported Ouctomes Measurement Information System Social Domain Working Group (2010). Measuring participation: The Patient-Reported Outcomes Measurement Information System experience. Arch Phys Med Rehabil.

[25] Nagl M, Gramm L, Heyduck K, Glattacker M, Farin E. Development and psychometric evaluation of a German version of the PROMIS® item banks for satisfaction with participation (2013). Evaluation and the Health Profession. Online first: http://dx.doi.org/10.1177/0163278713503468 10.1177/0163278713503468 24072786

[26] Fairbank JC, Pynsent PB (2000). The Oswestry Disability Index. Spine.

[27] Mannion AF, Junge A, Fairbank CT, Dvorak J, Grob D (2006). Development of a German version of the Oswestry Disability Index. Part 1: cross-cultural adaptation, reliability, and validity. Eur Spine J.

[28] Tait RC, Chibnall JT (2005). Factor structure of the pain disability index in workers compensation claimants with low back injuries. Arch Phys Med Rehabil.

[29] Dillmann U, Nilges P, Saile H, Gerbershagen H (1994). Behinderungseinschätzung bei chronischen Schmerzpatienten [Assessing disability in chronic pain patients]. Schmerz.

[30] Zigmond AS, Snaith RP (1983). The Hospital Anxiety and Depression Scale. Acta Psychiatr Scand.

[31] Herrmann-Lingen C, Buss U, Snaith RP (2005). HADS-D: Hospital Anxiety and Depression Scale - Deutsche Version: Deutsche Adaptation der Hospital Anxiety and Depression Scale.

[32] Glattacker M, Meixner K, Farin E, Jäckel WH (2007). Entwicklung eines rehabilitationsspezifischen Komorbiditätsscores und Prüfung der methodischen Gütekriterien [Development and psychometric testing of a rehabilitation specific comorbidity score]. Physikalische Medizin, Rehabilitationsmedizin, Kurortmedizin.

[33] Yuan YC (2000). Multiple imputation for missing data: Concepts and new development.

[34] Mayer B, Muche R, Hohl K. (2012). Software for the handling and imputation of missing data - An overview. J Clin Trials. 2

[35] Rubin DB (1987). Multiple imputation for nonresponse in surveys.

[36] Cole DA, Maxwell SE (2003). Testing mediational models with longitudinal data: Questions and tips in the use of structural equation modeling. J Abnorm Psychol.

[37] Anderson SE, Williams LJ (1992). Assumptions about unmeasured variables with studies of reciprocal relationships: The case of employee attitudes. J Appl Psychol.

[38] Bollen KA (1989). Structural equations with latent variables.

[39] Hu LT, Bentler P (1999). Cutoff criteria for fit indices in covariance structure analysis: conventional criteria versus new alternatives. Struct Equat Model.

[40] Tucker L, Lewis C (1973). A reliability coefficient for maximum likelihood factor analysis. Psychometrika.

[41] Farin E, Nagl M (2013). The patient-physician relationship in patients with breast cancer: descriptive results and influence on quality of life after rehabilitation. Qual Life Res.

[42] Cheung GW, Rensvold RB (2002). Evaluating goodness-of-fit indexes for testing measurement invariance. Struct Equat Model.

[43] Lindwall M, Larsman P, Hagger MS (2011). The reciprocal relationship between physical activity and depression in older European adults: A prospective cross-lagged panel design using SHARE data. Health Psychol.

[44] Weston R, Gore PA (2006). A Brief Guide to Structural Equation Modeling. Counsel Psychol.

[45] Sturgeon J, Zautra A (2013). State and trait pain catastrophizing and emotional health in rheumatoid arthritis. Ann Behav Med.

[46] Foster NE, Thomas E, Bishop A, Dunn KM, Main CJ (2010). Distinctiveness of psychological obstacles to recovery in low back pain patients in primary care. Pain.

[47] Wertli MM, Burgstaller JM, Weiser S, Steurer J, Kofmehl R, Held U (2014). Influence of catastrophizing on treatment outcome in patients with nonspecific low back Pain: A systematic review. Spine.

[48] Picavet HS, Vlaeyen JWS, Schouten JSAG (2002). Pain Catastrophizing and Kinesiophobia: Predictors of Chronic Low Back Pain. Am J Epidemiol.

[49] Burton AK, Tillotson KM, Main CJ, Hollis S (1995). Psychosocial predictors of outcome in acute and subchronic low back trouble. Spine.

[50] Wade JB, Riddle DL, Thacker LR (2012). Is pain catastrophizing a stable trait or dynamic state in patients scheduled for knee arthroplasty?. Clin J Pain.

[51] Crombez GP, Eccleston CP, Van Damme SP, Vlaeyen JWS, Karoly PP (2012). Fear-Avoidance Model of Chronic Pain: The next generation. Clin J Pain.

[52] Vlaeyen JWS, Linton SJ (2000). Fear-avoidance and its consequences in chronic musculoskeletal pain: a state of the art. Pain.

[53] Sturgeon JA, Zautra AJ (2013). Psychological resilience, pain catastrophizing, and positive emotions: perspectives on comprehensive modeling of individual pain adaptation. Curr Pain Headache Rep.

[54] Terwee CB, van der Slikke RMA, van Lummel RC, Benink RJ, Meijers WGH, de Vet HCW (2006). Self-reported physical functioning was more influenced by pain than performance-based physical functioning in knee-osteoarthritis patients. J Clin Epidemiol.

[55] Williams ACDC, Eccleston C, Morley S (2012). Psychological therapies for the management of chronic pain (excluding headache) in adults. Cochrane Database Syst Rev.

[56] Thorn BE, Boothby JL, Sullivan MJL (2002). Targeted treatment of catastrophizing for the management of chronic pain. Cognit Behav Pract.

